# Identification of the Features of Emotional Dysfunction in Female Individuals With Methamphetamine Use Disorder Measured by Musical Stimuli Modulated Startle Reflex

**DOI:** 10.3389/fnhum.2018.00230

**Published:** 2018-06-05

**Authors:** Xi-Jing Chen, Chun-Guang Wang, Wang Liu, Monika Gorowska, Dong-Mei Wang, Yong-Hui Li

**Affiliations:** ^1^CAS Key Laboratory of Mental Health, Institute of Psychology, Beijing, China; ^2^Department of Psychology, University of Chinese Academy of Sciences, Beijing, China; ^3^Beijing Municipal Bureau of Drug Rehabilitation Administration, Beijing, China

**Keywords:** substance use disorders, methamphetamine, music stimuli, startle reflex, females

## Abstract

Emotional dysregulation contributes to the development of substance use disorders (SUDs) and is highly associated with drug abuse and relapse. Music as a contextual auditory stimulus can effectively stimulate the reward circuitry, modulate memory associated with drug taking, and enhance emotional experiences during drug taking. However, the studies of the emotional responses to music in individuals with SUDs are scarce. Using startle reflex and self-reports, this study assessed the psychophysiological and cognitive emotional responses (i.e., valence, arousal and proximity) to happy, peaceful, and fearful music stimuli in 30 females with methamphetamine use disorder (MUD) and 30 healthy females. The results found that participants with MUD showed an inhibited startle response to fearful music compared to normal controls (*t* = 3.7, *p* < 0.01), and no significant differences were found in the startle responses to happy and peaceful music between the two groups. For the self-reported ratings, participants with MUD showed a decreased arousal in the response to fearful (*t* = 4.1, *p* < 0.01) and happy music (*t* = 3.8, *p* < 0.01), an increased valence in the response to fearful music (*t* = 4.4, *p* < 0.01), and a higher level of proximity in the response to fearful (*t* = 3.8, *p* < 0.01) and happy music (*t* = 2.2, *p* = 0.03). No significant differences were found in the rating of arousal to peaceful music, the valence to happy and peaceful music, as well as the proximity to peaceful music between the two groups. The females with MUD showed attenuated psychophysiological response and potentiated cognitive response (i.e., valence, arousal) to fearful music, as well as a high proximity to musical stimuli with high arousal regardless of its valence. These results have important implications for promoting the effectiveness of assessment and therapy selections for female MUD patients with impaired emotion regulation.

## Introduction

Substance use disorders (SUDs) is featured as “a cluster of cognitive, behavioral and physiological symptoms indicating that the individual continues using the substance despite significant substance-related problems” in Diagnostic and Statistical Manual of Mental Disorders Fifth Edition ([DSM-5], p. 483, American Psychiatric Association, 2013). Emotional dysregulation is an important problem that contributes to the development of SUDs (London et al., 2004, 2015). Chronic drug abuse involves the plastic change in the neural circuits mediating the reward system and anti-reward system (Koob and Moal, 2005). With the prolongation of drug use, the reward system increases its threshold (i.e., decrease neurotransmitter function) as a neuroadaptive change to make abusers become more difficult to experience pleasure feelings, and the anti-reward system including corticotrophin-releasing factor, norepinephrine and dynorphin are activated to produce negative or stress states. Consequently, depression and anxiety become the two most prevalent negative emotions in methamphetamine abusers (London et al., 2004). Moreover, research found the hypoactivation of the ventromedial prefrontal cortex/anterior cingulate cortex (vmPFC/rACC) and abnormal (i.e., no activation, hypoactivation, or hyperactivation) activation of amygdala and insula in individuals with SUDs (Salloum et al., 2007; Gilman and Hommer, 2008; O’Daly et al., 2012; Wilcox et al., 2016). These findings indicate the dysfunctional emotion regulation in people with SUDs, including dampened cognitive function for inhibiting intense affect, and abnormal emotion processing and reactivity.

Emotional reactivity has been commonly applied as one dimension for assessing the impairment of emotional regulation in people with SUDs (Blanchart et al., 2008; Smoski et al., 2011; Savvas et al., 2012). Previous studies that utilized visual emotional stimuli (i.e., pictures, facial expressions, videos) for eliciting emotional responses found inconsistent findings across the different types of drug users. Stimulant substance users (e.g., cocaine) demonstrated a more sensitive perception to the pleasant to unpleasant stimuli, and depressant users (e.g., heroin) tended to neutralize the rating on both unpleasant and pleasant stimuli (Kornreich et al., 2003; Aguilar de Arcos et al., 2005). These results suggest that there are different characteristics of emotional experience across drug types depending on the various clinical impact of drugs. In addition, research found the gender differences in emotion regulation between female and male with SUDs (Potenza et al., 2012). Compared to male cocaine dependents, female dependents showed an increased activation in amygdala and insula during a personalized stressful narrative, indicating that female with SUDs may be more vulnerable and experience more emotion regulation difficulties when facing stress. These findings suggest that gender should be taken into consideration in the assessment and therapy selection for treating people with SUDs.

The emotional reactivity can be measured using self-reports and psychophysiological measurements. The self-reported valence, arousal and proximity of emotions assess the cognitive aspect of emotion regulation (i.e., emotion recognition, perception). Valence refers to the nature of the emotional stimulus (i.e., positive vs. negative, or pleasurable vs. unpleasant); arousal refers to the intensity of the stimulus (i.e., low or high intensity); and proximity refers to the motivational reaction toward the emotional stimulus (i.e., approach or avoidance). Valence and arousal reflect the nature and intensity of motivational activation respectively (Bradley et al., 2001). The research found that individuals with SUDs reported high arousal (i.e., increase of anxiety, heart rate and salivary cortisol levels), negative valence (increased negative emotion), and avoidance motivation in the response to stressful stimuli, which often lead to drug craving and abuse (Sinha et al., 2000; Baker et al., 2004). Psychophysiological measurements mainly focus on assessing the implicit physiological responses to emotions with or without consciousness. Startle reflex is an effective measure that has been extensively used for probing emotional reactivity (Lang et al., 1990; Cook et al., 1992). As a response of the defensive emotional system, it can record the automatic defensive reaction (i.e., the amplitude of the eye link) in response to a loud white noise. The startle reflex is enhanced in response to negative emotional stimuli and is inhibited in response to positive emotional stimuli in normal people (Lang et al., 1998; Bradley and Lang, 2000).

Music as auditory stimuli can effectively modulate emotional experience. Music reward involves the brain regions that highly overlap with the regions of drug reward (Salimpoor et al., 2011; Zatorre and Salimpoor, 2013). A fMRI study (Menon and Levitin, 2005) found that music mediated the activity of mesolimbic reward circuitry including nucleus accumbens (NAcc), ventral tegmental area (VTA), hypothalamus and insula. Pleasant music significantly activated the interaction between the NAcc and hypothalamus, as well as insula and orbitofrontal cortex (Blum et al., 2010). The quality of musical elements (e.g., rhythm, harmony, timbre, musical structure, speed, power and melody) is associated with the valence and arousal of emotional experiences (Zhou, 1999). It is important to note the distinct characteristics of music as an emotional stimulus comparing to other kinds of emotional stimuli (e.g., picture, video, script). For example, people normally withdraw or avoid from the negative emotional stimuli, yet some listeners have an approach motivation toward sad music that match their affect state for improving mood (Garrido and Schubert, 2013).

Moreover, musical experience and training can change the plasticity of brain regions related to emotion regulation. Musicians and people with musical training exhibited a higher level of musical rewarding experience than people with no musical background (Mas-Herrero et al., 2013). A EEG study revealed that after 3 months of improvisational music therapy for depressed clients, significant increased absolute power was found at left fronto-temporal alpha and theta, indicating the impact of music intervention on reducing depression and anxiety symptoms (Fachner et al., 2013). Gender effect was found when use music for emotion regulation. The activation of medial prefrontal cortex (mPFC) decreases in males and increases in females during music listening (Carlson et al., 2015).

In the context of SUDs, animal study demonstrated that after repeatedly associated with methamphetamine, music as a contextual conditioned stimulus can significantly increase extracellular dopamine levels in the nucleus accumbens and basolateral amygdala, as well as locomotor activity in rats (Polston et al., 2011), suggesting that music can effectively stimulate the dopamine circuitry and modulate associated memory of drug taking. An investigation of 143 substance abusers found that music was a common contextual stimulus during drug using. Seventy percent of the substance abusers listened to music for more than 1 h each day, and reported that music enhanced their emotional experience during drug taking (Dingle et al., 2015).

Despite the powerful impact of music on emotion regulation, the studies of emotional responses to music in people with SUDs are scarce. Given the different neurotoxicity of drugs and gender effect, the aim of this study is to explore the emotional perception and responses to music stimuli in females with methamphetamine use disorder (MUD). We hypothesize that female individuals with MUD will have a biased emotional perception and response to pleasant and unpleasant musical stimuli compared to normal controls.

## Materials and Methods

### Participants

Thirty female participants with MUD were recruited from the Xin-He Drug Rehabilitation Center, and 30 healthy female participants as controls were recruited from a manufacture factory in Beijing, China. Two psychologists interviewed all participants for gathering demographic information and screening, and then participants filled out the State-Trait Anxiety Inventory, Beck Depression, and Barcelona Music Reward Questionnaire for assessing anxiety, depression and musical reward sensitivity.

For the MUD participants, the inclusion criteria are: (1) aged 18–55 years; (2) a history of using methamphetamine and fulfilled the diagnosis of stimulant use disorder in the Chinese version of DSM-5 (pp. 232–238, American Psychiatric Association, 2014). Stimulant use disorder refers to the clinically significant impairment or distress caused by the use of amphetamine-type substance, cocaine, or other kinds of stimulant, such as amphetamine, dextroamphetamine, methamphetamine, cocaine and methylphenidate.

Exclusion criteria: (1) a history of brain damage or a coma over 30 min; (2) a history of using other kind of drugs (e.g., heroin, cocaine); (3) illiteracy; (4) a history or a family history of mental illness; and (5) hearing problems. All participants had no musical training history. The study was carried out in accordance with the recommendation of the Declaration of Helsinki. The protocol was approved by the Ethics Committee of Institute of Psychology, CAS (H17001). All subjects gave a written informed consent in accordance with the Declaration of Helsinki.

### Musical Excerpts

Three musical excerpts presenting three emotions (i.e., happy, fearful, peaceful) were adapted from a previous study (Vieillard et al., 2008). Five excerpts of each emotion were selected out of a pool of 42 excerpts based on the assessment of 30 music majored colleague students. They evaluated the valence (0 = pleasant, 9 = unpleasant) and arousal (0 = relaxing, 9 = stimulating) of each musical excerpt using a Likert scale. The happy excerpts were selected based on the high arousal and valence, the fearful excerpts were selected based on the high arousal and low valence, and the peaceful excerpts were selected based on the low arousal and high valence. Then the top five excerpts of each emotion were selected for the study (see Supplementary Table S1 for the list of the music excerpts).

Fifteen chosen music excerpts were further evaluated and validated by 46 college students with no musical training on the dimensions of valence, arousal and proximity (0 = approach, 9 = withdraw). In terms of valence, happy excerpts was higher than peaceful excerpts (*t* = 5.6, *p* < 0.01) and fearful excerpts (*t* = 16.95, *p* < 0.01), and peaceful excerpts was higher than fearful excerpts (*t* = 12.25, *p* < 0.01); in terms of arousal, peaceful excerpts was lower than happy excerpts (*t* = −7.59, *p* < 0.01) and fearful excerpts (*t* = −5.45, *p* < 0.01), and there was no significant difference between happy and fearful excerpts; in terms of proximity, fearful excerpts was lower than peaceful excerpts (*t* = −16.69, *p* < 0.01) and happy excerpts (*t* = −14.02, *p* < 0.01), and no significant difference was found between happy and peaceful excerpts. The results indicated that the selected music excerpts elicited differentiated emotions corresponding with their valence, arousal, and proximity. All music excerpts were piano melodic music produced by a digital synthesizer with a duration from 10 s to 14 s. Each excerpt was normalized to equate loudness using the normalization function of the *Audition 3* software.

### Measurements

#### Startle Reflex

**Figure 1:**
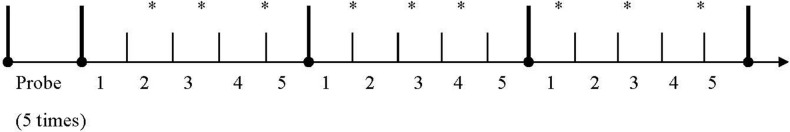
Distribution of probes during the presentation of three types of music excerpts. *Note*: *White noise probe.

White noise of 100 dB, 50 ms burst was presented over Sony MDR-XB500AP head-phones to elicit startle responses. Five white noise probes were presented during the first minute with a randomized interval before the presentation of music stimuli. Then three types of music excerpts were presented with a 3 s interval between each excerpt, and each type of music was presented with three randomly placed startle probes (Figure 1).

For startle data recording, the Eye-blink Electromyographic (EMG) data were collected from the orbicularis oculi using two mini-electrodes placed below the left eye (Larson et al., 2005). After each white noise, EMG activity (μv) was automatically recorded. EMG signals pass through bandpass filtered at 10 and 500 Hz and were amplified by 1000. The sampling rate was set at 1000 Hz. The maximum amplitude of each response between 20 ms and 120 ms after startle probe onset was considered as valid data and included for analysis (Balaban et al., 1986). To reduce individual variability in the raw startle reflex data, the raw data were standardized within in each participant, In the light of a previous study (Roy et al., 2009), the standardized score was expressed as T scores (50 + 10 Hz), which led to a mean of 50 and a standard deviation of 10 for each participant.

#### Self-Reported Emotional Responses

Self-reported emotional responses to music excerpt were measured using a Likert scale scored from 0 to 9. After each music excerpt, the participants assess it on the dimension of valence (0: unpleasant, 9: pleasant), arousal (0: relaxing, 9: stimulating), and proximity (0: withdraw, 9: approach).

#### Anxiety

Anxiety was measured using the Chinese version of State-trait Anxiety Inventory. It consists of 20 items and ranged from 20 to 80. Higher scores indicate higher level of anxiety.

#### Depression

Depression was measured using the Chinese version of Beck Depression Inventory. It consists of 13 items and ranged from 0 to 63 (4–7: mild depression, 8–15: moderated depression, 16 or higher: severe depression). Higher scores indicate higher level of depression.

#### Musical Reward Sensitivity

Barcelona Musical Reward Questionnaire was developed by Mas-Herrero and his colleagues (Mas-Herrero et al., 2013) for assessing music reward sensitivity. It evaluates the sensitivity to music from the dimensions of emotional evocation, sensory-motor, mood regulation, musical seeking and social reward. It consists of 20 items and scored from 1 (completely disagree) to 5 (completely agree). A higher score indicates a higher level of musical reward sensitivity (0–40: low, 40–60: standard, 60 or higher: high).

### Procedure

The researcher helped the participants to put on headphones and affix the electrodes for startle reflex. The participants sat comfortably in a quiet room and watched natural scenes (i.e., the sea life aquarium) on a computer screen for 1 min to relax. Before the presentation of the music stimuli, five white noise probes were played randomly during 1 min. Then, three types emotional music excerpts were presented in a counterbalanced order across subjects. The startle reflex responses were recorded during the listening process. After each music excerpt, the participants rated the valence, arousal and proximity of the excerpt.

### Data Analysis

Data analysis was performed using the Statistical Product and Service Solutions (SPSS) 17.0. The comparison of demographic information, anxiety, depression, music reward, self-reported ratings and startle reflex between two groups were analyzed using independent sample *t*-test.

## Results

The two groups were matched in age, anxiety, depression and musical reward sensitivity (Table 1). The control group had more years of education than the methamphetamine (MA) group.

**Table 1:** The comparison between the two groups in demographic information and clinical characteristics.

	Methamphetamine group (*n* = 30)	Control group (*n* = 30)	Difference *p*
Outcome	M (*SD*)	M (*SD*)	
Age (year)	30.97 (7.41)	29.58 (7.17)	0.09
Education (year)	9.21 (3.12)	12.30 (2.14)	0.01**
BMRQ	76.21 (9.08)	75.83 (7.31)	0.07
BDI	12.63 (9.69)	12.35 (9.35)	0.08
TSAI (state)	37 (10.38)	36.67 (9.56)	0.07
TSAI (trait)	39.64 (7.93)	40.21 (8.22)	0.06
Abstinent period (month)	8.68 (3.64)	-	-
Total time of drug use (month)	35.23 (22.41)	-	-
Total drug use amount in a year (gram)	82.35 (124.53)	-	-

Note. BMRQ, Barcelona Musical Reward Questionnaire; BDI, Beck Depression Inventory; STAI, State and Trait Anxiety Inventory; **p ≤ 0.01.

### Startle Reflex

**Figure 2:**
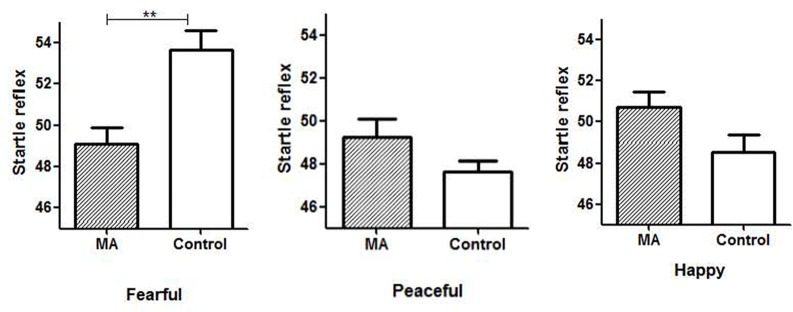
Startle reflex amplitudes in response to three emotional music excerpts in the two groups. *Note*: MA, methamphetamine group; Control, healthy control group; **p ≤ 0.01.

Compared to the normal control, the MA group showed a lower level of startle response to fearful music (MA: 49.08, Control: 53.66, *t* = 3.7, *p* < 0.01). There was no significant difference in the response to peaceful and happy music between the two groups, although the startle reflex amplitudes to both music stimuli in the MA group were higher (Figure 2).

### Self-Reported Emotions

**Figure 3:**
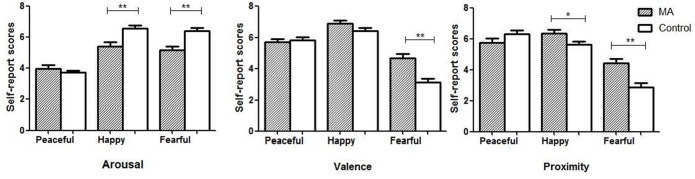
The self-reported emotions of arousal, valence and proximity in the two groups. *Note*: MA, methamphetamine group; Control, healthy control group; *p ≤ 0.05, **p ≤ 0.01.

Compared to the control group, the MA group showed a lower level of arousal in response to happy music (*t* = 3.8, *p* < 0.01) and fearful music (*t* = 4.1, *p* < 0.01), a higher valence (i.e., more pleasant) in response to fearful music (*t* = 4.4, *p* < 0.01), and a higher proximity (i.e., approach motive) in response to happy (*t* = 2.2, *p* = 0.03) and fearful music (*t* = 3.8, *p* < 0.01; Figure 3).

## Discussion

The female participants with MUD showed an inhibited startle response to negative (i.e., fearful) music, and a tendency of potentiated startle response to positive music (i.e., happy, peaceful). These reactivities that opposite to the reactions in normal people may indicate the impaired emotional processing and emotional regulation (Lang et al., 1998; Bradley and Lang, 2000). Moreover, the increased self-reported valence to fearful music, and decreased self-reported arousal of MUD participants to both positive and negative music accords with their dysfunctional startle reflex response, which reflects the dampened emotional perception on the emotional valence and arousal. These findings are in line with previous studies (Carrico et al., 2013; May et al., 2013) that people with SUDs show attenuated response to emotional stimuli.

The participants with MUD showed a higher level of valence and lower level of arousal, as well as a decreased startle response to fearful music than the normal controls suggesting their biased emotional perception and psychophysiological reactivity to music. Tempo and harmony influence the arousal and valence of music emotional experience respectively (Gomez and Danuser, 2007; Hodges, 2010). The fearful music excerpts used in the study feature fast tempo and dissonant melodies, which may create intense and stimulating feelings. The MUD participants also showed a higher level of proximity to both positive and negative music with high arousal. Given the chronic drug use impairs the reward system, individuals with dampened reward function may seek for the strong stimulant feature of high arousal music to acquire pleasurable feelings. Huron (2011) pointed out that the brain can distinguish “fake” negative emotions in music from real threat in life, therefore it is “safe” for people to enjoy music that conveys negative emotions. Drug abusers tend to use drugs to decrease or avoid negative feelings (Otto et al., 2004). To be open and experience “negative” music and may help them to face their negative feelings and deal with their problems instead of taking drugs. For music therapy, music with high arousal may be used to attract the attention and increase the motivation of patients with SUDs.

It is noteworthy that there was no significant difference in the cognitive responses including valence, arousal, and proximity to peaceful music between the two groups. Given depression and anxiety are the two most prominent negative emotions in methamphetamine users (London et al., 2004), this result may suggest the suitability of using peaceful music for relaxation in treating patients with MUD as they may respond well to peaceful music. In addition to music listening, active musical activities such as improvisation can help people to explore and express various feelings, facilitate meaningful communicate, gain public recognition and bring a sense of achievement (Soshensky, 2001; Baker et al., 2007; Silverman, 2009).

The study has several limitations. Only female subjects with MUD participated in this study, there is a lack of the comparison between two genders. The educations years in participants with MUD are less than the healthy controls. However, the music listening task did not require a high level of cognitive function, therefore we suppose this difference did not affect the task. The future study will add male participants, improve the comparability of the two groups, and supply more psychophysiological measurements, such as ERP, EEG, skin conductance.

In summary, the study utilized emotional music stimuli to elicit emotional responses of female individuals with MUD, and assessed them with cognitive and psychophysiological measurements. The results found that the females with MUD showed inhibited psychophysiological and cognitive emotional responses to fearful music, and a high proximity to musical stimuli with high arousal regardless of its valence. These results have important implications for promoting the effectiveness of assessment and therapy for female MUD patients with impaired emotion regulation.

## Datasets Are Available on Request

The raw data supporting the conclusions of this manuscript will be made available by the authors, without undue reservation, to any qualified researcher.

## Author Contributions

X-JC designed and implemented the experiment, analyzed the data and drafted the manuscript. C-GW and WL conducted the interview for the participants. MG helped with the data collection process. D-MW and Y-HL guided the study design and directed the experiment implementation.

## Conflict of Interest Statement

The authors declare that the research was conducted in the absence of any commercial or financial relationships that could be construed as a potential conflict of interest.

## Abbreviations

MUD: methamphetamine use disorder
SUDs: substance use disorders.
