# Supplementary material for: Identification of the Features of Emotional Dysfunction in Female Individuals With Methamphetamine Use Disorder Measured by Musical Stimuli Modulated Startle Reflex
Source: Front Hum Neurosci. 2018 Jun 5;12:230. doi: 10.3389/fnhum.2018.00230 (PMC5996031; doi:10.3389/fnhum.2018.00230)
Supplement: Supplementary file 1 [file Table_1.DOCX]

**Supplementary materials**

**Table 1. The music excerpts used in the study**

| Music excerpt | Number | Number | Number | Number | Number |
| --- | --- | --- | --- | --- | --- |
| Peaceful | a03 | a11 | a14 | a10 | a09 |
| Happy | g08, | g02 | g04 | g10 | g07 |
| Fearful | p14 | p06 | p03 | p08 | p01 |

*Note:* The designations of the excerpts are the same designations

(The order of the excerpts was based on the ranking of their valence and arousal by 46 college students with no musical training.)

The music excerpts were adapted from:

Vieillard, S., Peretz, I., Cosselin, N., Khalfa, S., Gagnon, L., & Bouchard, B. (2008). Happy, sad, scary and peaceful musical excerpts for research on emotions. *Cognition and emotion, 22*(4), 720-752.
